# Current trends in the management of extra-abdominal desmoid tumours

**DOI:** 10.1186/1477-7819-4-21

**Published:** 2006-04-03

**Authors:** Panayiotis J Papagelopoulos, Andreas F Mavrogenis, Evanthia A Mitsiokapa, Kleo Th Papaparaskeva, Evanthia C Galanis, Panayotis N Soucacos

**Affiliations:** 1 First Department of Orthopaedic Surgery, Athens University Medical School, Athens, Greece; 2 Department of Physical Medicine and Rehabilitation, Thriassion Hospital, Elefsis, Greece; 3 Department of Pathology, Hygeia Athens Medical Center, Athens, Greece; 4 Department of Medical Oncology, Mayo Clinic and Mayo Foundation, Rochester, Minnesota

## Abstract

Extra-abdominal desmoid tumours are slow-growing, histologically benign tumours of fibroblastic origin with variable biologic behaviour. They are locally aggressive and invasive to surrounding anatomic structures. Magnetic resonance imaging is the modality of choice for the diagnosis and the evaluation of the tumours. Current management of desmoids involves a multidisciplinary approach. Wide margin surgical resection remains the main treatment modality for local control of the tumour. Amputation should not be the initial treatment, and function-preserving procedures should be the primary treatment goal. Adjuvant radiation therapy is recommended both for primary and recurrent lesions. Chemotherapy may be used for recurrent or unresectable disease. Overall local recurrence rates vary and depend on patient's age, tumour location and margins at resection.

## Background

Extra-abdominal desmoid tumours or aggressive fibromatoses are rare, slow-growing, histologically benign tumours of fibroblastic origin, with variable biological behaviour [1-3]. Desmoid tumours account for about 0.03% of all neoplasms, and less than 3% of all soft tissue tumours. The estimated incidence in the general population is 2 to 4 per million population per year [4].

Despite their benign appearance, they are locally aggressive and invasive to surrounding anatomic structures. Because of these characteristics, desmoid tumours are often classified as low-grade fibrosarcomas [1,2,5,6].

Although aetiology is unknown, a connective tissue growth disorder has been supported [7-10]. Desmoid tumours have been associated with hereditary syndromes (Gardner's syndrome), pregnancy, especially second pregnancy, and endogenous/exogenous female sex hormones in adults. However in children, these associations are difficult to establish [4,10,11].

Based on X-chromosome inactivation pattern, desmoid tumours appear to be monoclonal disorders, indicative of a neoplastic rather than an inflammatory fibrous reactive process [5,8,13]. Other studies [7,9] have reported an abnormal expression of c-sis and platelet-derived growth factor (PDGF), which can be mitogen for fibrocytes, and a somatic APC gene mutation in patients with increased levels of beta-catenin, which may explain the proliferative advantage of these cells. Besides loss-of-function mutations of APC, gain-of-function mutations of beta-catenin have also been described [9,14]. In addition, the tumour-suppressor gene Rb1 has shown decreased expression and may also play a role in the pathophysiology of the disease [10].

### Location

Desmoid tumors are fibroproliferative disorders arising within musculoaponeurotic tissue, observed in nearly every part of the body. Numerous sites in the trunk and extremities have been identified, and two forms of the disease are distinguished; an extra-abdominal and an intra-abdominal form. Generally, desmoids usually occur in three locations; the extremities (commonly around the limb girdles or the proximal extremities); the abdominal wall (commonly found in women, especially during and after pregnancy); and the bowel wall and mesentery (often associated with Gardner syndrome) [5,15].

In approximately 10% of the cases, multicentric disease has been reported [16,17]. Multicentricity is defined as the presence of disease in more than one location at the time of diagnosis. Multicentric lesions tend to be in the same limb or anatomic region, and it is possible that many cases of distant or local recurrence of primary lesions within the same region represent multicentric disease that were not clinically apparent at the initial diagnosis [4,16-19].

### Natural history

The clinical behaviour and natural history of desmoid tumours remain unpredictable. They are usually slow-growing, locally aggressive and invasive to surrounding tissues. Although both spontaneous regression and disappearance have been reported, as has spontaneous regression after biopsy [2,5,20,21], most lesions will progress refractory to multiple surgical procedures and adjuvant therapy. They often recur after surgery, particularly after marginal or intralesional excision. Distant haematogenous or lymph nodes metastases have not been observed, and multicentric disease and recurrence or reactivation at sites other than the primary location have been reported [16,17]. Extra-abdominal head and neck lesions are more aggressive than extra-abdominal desmoids arising elsewhere and are capable of massive destruction of adjacent bone and erosion of the base of the scull, and occasionally encroach on the trachea, some times with fatal outcome. [2,19,22-24]. Loss of function may be observed with extra-abdominal tumours involving the extremities, as result of wide resection, local recurrence, and radiation therapy [2,5].

### Imaging

**Figure 1:**
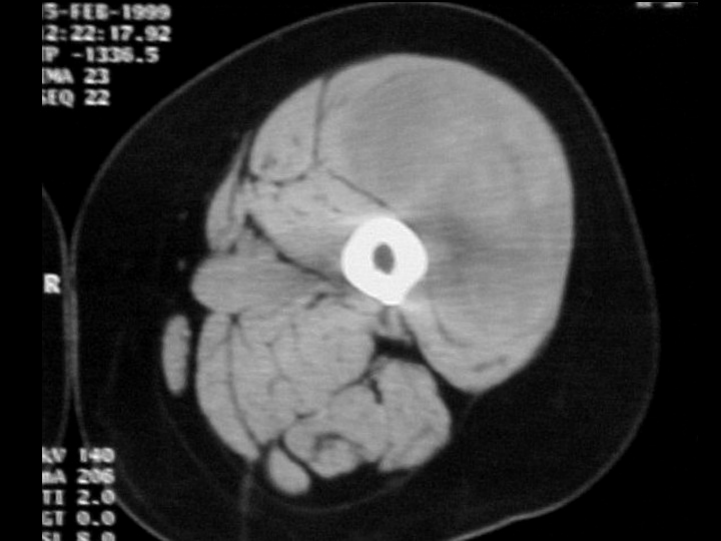
Computed tomography scan of the left thigh shows a large soft tissue mass involving the vastus lateralis muscle.

**Figure 2:**
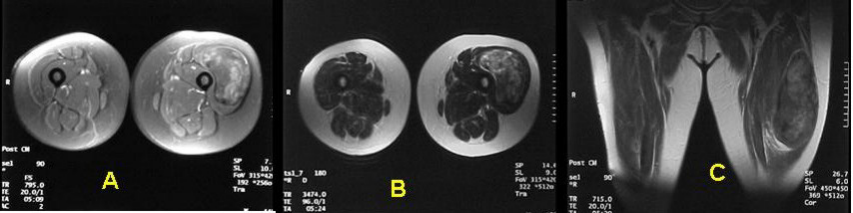
Magnetic tomography imaging **(A) **axial T1-weighted, **(B) **T2-weigthed, and **(C) **coronal T1-weighted views show a well-circumscribed heterogeneous soft tissue mass within the left vastus lateralis muscle.

Although computed tomography shows the extent of the tumour and its relationship to the neurovascular structures (Figure 1), magnetic resonance imaging (MRI) is the modality of choice for the diagnosis and the evaluation of the tumours' extent and the progression of the disease after treatment (Fig 2). MRI features vary. Heterogeneous changes are common, depending on the distribution of collagen and the cellularity of the lesions [25,26]. The lesions may be hypo- or hyper-intense relative to surrounding muscle or adipose tissue (Fig 2B,C), on both T1- and T2-weighted sequences, with both sharply and poorly defined margins [26,27].

MRI with gadolinium injection may also be helpful in differentiating tumour progression from post-surgical fibrosis [26]. Multicentric and recurrent lesions tend to occur within the same limb or anatomic region, which outlines the role of scanning the entire extremity once the diagnosis is made [17].

### Pathology

**Figure 3:**
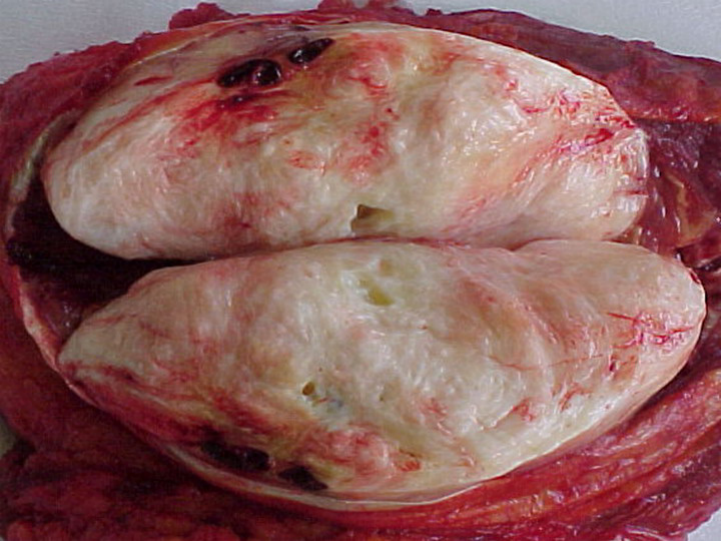
Photograph of the gross specimen excised shows a compact fibrous and lobulated soft tissue tumour.

On gross examination, the tumours are always confined to the musculature and the overlying aponeurosis or fascia (Figure 3). Their size varies from 5 to 20 cm. The tumours are firm, cut with a gritty sensation, and on cross section reveal a glistening white, coarsely trabeculated surface resembling scar tissue [24].

**Figure 4:**
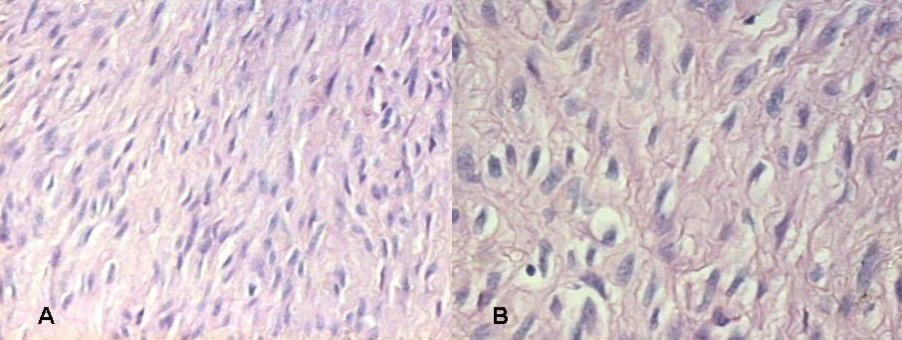
Photomicrographs in **(A) **low-power and **(B) **high-power show elongated, slender, spindle-shaped cells of uniform appearance without atypia. The cells are surrounded and separated from one another by abundant collagen, with little to no cell-to-cell contact.

Microscopically, desmoid tumours are poorly circumscribed, infiltrating the surrounding tissue. The proliferation consists of elongated, slender, spindle-shaped cells of uniform appearance. The tumour cells are surrounded and separated from one another by abundant collagen, with little to no cell-to-cell contact (Figure 4A,B). The cells lack atypia, but cellularity may vary within the same lesion. Nuclei are small, pale-staining, and sharply defined. One to three small nucleoli are usual [24,28].

Ultrastructurally, desmoid tumours consist of a uniform population of elongated fibroblast-like cells, often terminating in long, slender processes [24,29]. Most nuclei are rounded or oval, but some cells show prominent nuclear indentations or clefts. There is a prominent rough endoplasmic reticulum, partly dilated, containing granular or fibrillary material within the dilated spaces. The cytoplasm has a small number of mitochondria, a prominent Golgi apparatus, free ribosomes, and occasional pinocytotic vesicles and microtubules. Some cells contain intracytoplasmic bundles of actin-type microfilaments, and incomplete or clumped basal lamina along the cell borders, all features characteristic of myofibroblasts. The stroma contains considerable amounts of collagen and ground substance [24,29].

### Differential diagnosis

Differential diagnosis includes fibrosarcoma, reactive fibroblastic proliferations, desmoplastic fibroma, myxoma, and nodular fasciitis [24,30].

Desmoids most closely resemble fibrosarcomas. An inadequate biopsy specimen may lead to misdiagnosis, as some examples of fibrosarcomas have areas indistinguishable from desmoid tumours and vice versa [24]. Desmoids may also be difficult to be distinguished from reactive fibroblastic proliferations following injuries such as trauma, minor muscle tears, or intramuscular injections. Cytologically, these reactive proliferations are composed of cells indistinguishable from those found in desmoid tumours, and the low-magnification appearance is more useful for differential diagnosis of these two entities. Desmoplastic fibroma of bone may also be indistinguishable from desmoid tumours, especially when presents as a soft tissue mass after breaking through the thinned or expanded cortex of the involved bone. Confusion with myxoma or nodular fasciitis is possible, particularly if only a small amount of tissue or FNA is available for examination [24].

Fibrosarcomatous transformation of desmoid tumours is rare, and it may be erroneously suggested by occasional foci of increased cellularity and by exceptionally well-differentiated fibromatosis-like areas in some fibrosarcomas [24].

### Management

Current management of desmoid tumours involves a multidisciplinary approach. Radiation therapy or brachytherapy have been used in the adjuvant setting. Antiestrogens, goserelin, progesterone, non-steroidal anti-inflammatory drugs (NSAIDs), interferon (IFN-alpha), cytotoxic chemotherapeutic agents, and radiation therapy have all been employed in the management of desmoids [1,2,4,5,31-41].

#### Surgical treatment

Wide margin surgical resection remains the main treatment option for local control of the tumour in all age groups [3,18,19,21,22,24,39,42,43]. Although often difficult to achieve because of the tumour's extent and invasiveness, wide margin surgical resection offers the best chance of avoiding local recurrence [3,19,21,24,42,44]. Desmoids frequently recur after surgery, and the benefits of radical surgery and various adjuvants must be weighed against the potential morbidity of the tumour, especially in the skeletally immature patients. The rate of surgical complications, such as joint contractures and poor function is also high [21].

Because of the infiltrative nature of these tumours, local recurrence rates after wide local excision may be high. Although several authors have reported a significantly increased risk of local recurrence in patients with positive or close resection margins, and intralesional or marginal excision, others have found no differences in local recurrence rates between patients with positive or negative margins [5,6,21,22,42-46]. Thus, the precise association of the surgical resection margins with local recurrence risk is difficult to evaluate [4,21,22,45,47,48]. Re-operation for the treatment of recurrent disease is advocated by most authors, resulting in a cure rate similar to that of the primary surgical resection [2,4,5,22]. Amputation should not be a primary treatment. It is considered inevitable and should be reserved for cases in which either recurrent disease or the effects of surgical treatment or irradiation have resulted in a significant loss of function and/or chronic symptoms [5,49].

### Radiation therapy

External-beam radiation therapy has been shown to improve local control of desmoid tumours, both in the adjuvant and primary settings [5,6,21,46-48]. It has been used as adjuvant mainly in adults for the treatment of unresectable or inaccessible disease, for gross residual tumour with positive or equivocal surgical margins, and to avoid mutilating surgery [6,19,21,22,42,46-48,50,51]. Radiation doses of 50 to 60 Gy are currently suggested as curative [2,5,6,22,42,44,46,47]. Lower doses are associated with higher rates of tumour progression and local recurrence. Higher doses have resulted in complications, including fibrosis, oedema, skin ulceration, neurological deficits including paresthesias and paresis, pathologic fractures, cellulitis, and secondary malignant transformation, which may also lead to a second stage amputation [42,47-49,52,53].

When used as adjuvant, irradiation is reported to achieve disease control in 77 to 90% of patients [6,12,42,54]. In cases of positive surgical margins, adjuvant radiation therapy is recommended when the potential morbidity of a second operation is high [5,6,12,42,44,45,50,54,55]. Intralesional excision in addition to radiotherapy is ineffective, with high local recurrence rates and life threatening complications for the patient. Under these circumstances doses above 50 Gy are recommended [2,12].

In children, desmoid tumours are highly aggressive, and data are not in favour of the use of irradiation because of the previously mentioned complications and the risks of contractures development and growth disturbance [18,19,40,52,53]. Most children are treated with radiation therapy using guidelines outlined for adults. External beam radiation therapy in children should be performed as a last resort, in patients reaching skeletal maturity, in cases with potential mortality or significant morbidity, and in tumours showing active growth adjacent to vital structures or progressive disease after multiple attempts at control with surgery and chemotherapy. Otherwise, when safe surgical margins can be achieved, no additional therapy is recommended, despite a risk of recurrence ranging from 5% to 50% [4,12,18,19,54].

### Management of recurrent or inoperative lesions

The role of radiation therapy is discussed above. Because of its low toxicity endocrine therapy is frequently the first line of treatment for recurrent desmoid tumours, which are not candidates for surgical or radiation approaches. The use of hormonal therapy for the treatment of these tumours is based on the association of oestrogen levels with the growth of desmoid tumours, and on estrogen receptor expression in desmoids [56,57]. A higher incidence of these tumours, especially in the abdominal wall, is reported during or soon after pregnancy, or during treatment with oral contraceptives [37,58,59]. There have also been reports of spontaneous regression of desmoids after menopause and oophorectomy [4,45]. Endocrine treatment with agents such as tamoxifene have resulted in response rates of up to 50% in older series [38]. Other hormonal agents employed in the treatment of desmoids include medroxyprogesterone acetate, megace, toremifen, and gonadotropin releasing hormone analog goserelin in tamoxifen failures

Non-steroid anti-inflammatory drugs such as indomethacin or sulindac as single agents or in combination with antioestrogens [60,61] have also been used in the treatment of desmoid tumours, with variable response rates being reported mostly based on small series and case reports [5,37].

Use of IFN-alpha in desmoids has also been reported in inoperable cases [62], and in combination with tretinoin in order to delay of local recurrence. Without clear evidence for long term benefits however, IFN-alpha therapy because of its relative lack of toxicity, it might be appropriate to consider in cases where mutilating surgery or more toxic medical treatment would be the other treatment alternative [1,32,34,35,45,63-65].

#### Chemotherapy

Cytotoxic chemotherapeutic agents have been used in inoperable or unresectable tumours, progressive or residual disease, and in rare cases as neoadjuvant to facilitate wide surgical resection. Although their histological appearance might suggest otherwise, desmoid tumours do respond to chemotherapy with symptomatic relief and occasionally durable disease regression. In children, combination chemotherapy may be indicated for inaccessible disease or in cases in which local control could not be achieved [5,18,32,37,52,53,59,60,66,67].

Low dose combination of vinblastine and methotrexate, vincristine, actinomycin D, cyclophosphamide, and doxorubicin/dacarbazine [2,66] have been used for symptomatic relief, tumour growth retardation in children in order to delay radiation therapy until skeletal maturity [5,66,68-70]. In one trial of 30 patients, weekly vinblastin and methotrexate resulted in a partial response rate of 40% and disease stability in 60% of patients. After a median follow-up of 75 months the 10 year actuarial progression free interval was 67% [71]. In another study of 7 patients treated with combinations of cyclophosphamide and doxorubicin, mitomycin, and etoposide objective regression was observed in 3 patients with duration of benefit ranging from 3 months to 15 years [49]. Finally in familiar adenomatous polyposis patients with desmoid tumours, a doxorubicin/dacarbazine combination followed by treatment with COX-2 inhibitor meloxicam resulted in objective responses in all 7 patients treated with a median progression free survival of 74 months. [72]

### Prognosis

Although neither haematogenous nor lymphatic metastases have been observed, most lesions will progress refractory to treatment. Spontaneous regression and disappearance, regression after biopsy, multicentric disease, and recurrence or reactivation at sites other than the primary location have all been reported [2,5,20,21]. Mortality is rare in extra-abdominal desmoid tumours, but has been reported in head and neck lesions. Loss of function is often observed with involvement of the extremities [19,22,23].

Overall local recurrence rates range from 19% to 75% [4,18,21,22,24,42,51]. Most recurrences are usually observed within 3 years, and nearly all by 6 years [2,18]. Age may affect recurrence rates, and although unclear, local recurrence may be more likely in younger patients with extra-abdominal lesions [2,4]. The region of involvement also affects local recurrence, which is reported higher in extra-abdominal lesions of the foot and calf. Extremity tumours, because of their locally aggressive behaviour have the higher propensity for local recurrent growth [5]. Local recurrence rates for intra-abdominal tumours are higher than those reported for extra-abdominal tumours, ranging from 57% to 86% after complete resection, with a higher complications rate and even death [5,15].

## Conclusion

Desmoid tumours are challenging with unpredictable natural history. Current management involves a multidisciplinary approach. Wide margin surgical resection remains the main treatment modality for local control of the tumour in all age groups. Amputation should not be a primary treatment and should be reserved for refractory cases or for those in whom the disease or its treatment have resulted in a non-functional or chronically painful extremity. Function and structure-preserving procedures should be the primary treatment goal.

Non-surgical treatment involving irradiation, brachytherapy, endocrine therapy and cytotoxic chemotherapy has resulted in diverse and unpredictable outcome. External-beam radiation therapy can be used as adjuvant mainly in adults, while in the skeletally immature patients should be used as a last resort in selected cases. Combination chemotherapy may be indicated for unresectable disease.

Overall local recurrence rates vary, depending on patient's age, tumour location and margins at resection. Disease progression may occur many years after treatment, and patients should be constantly surveyed. Future research efforts forwards development of targeted therapeutics may improve patients' outcome.

## Competing interests

The author(s) declare that they have no competing interests.

## Authors' contributions

**PJP **searched literature, provided the figures, drafted the manuscript and edited the final version, **AFM **assisted in manuscript preparation, **EAM **assisted in manuscript preparation, **KTP **carried out the histopathological studies of presented case and drafted the manuscript on the section of pathology, **ECG **provided referral for nonoperative treatments, and drafted the manuscript, **PNS **drafted the manuscript and edited the final version.

All authors read and approved the final manuscript.
